# Polyurethane/*n*-Octadecane Phase-Change Microcapsules via Emulsion Interfacial Polymerization: The Effect of Paraffin Loading on Capsule Shell Formation and Latent Heat Storage Properties

**DOI:** 10.3390/ma16196460

**Published:** 2023-09-28

**Authors:** Denis V. Voronin, Eliza Sitmukhanova, Rais I. Mendgaziev, Maria I. Rubtsova, Dmitry Kopitsyn, Kirill A. Cherednichenko, Anton P. Semenov, Rawil Fakhrullin, Dmitry G. Shchukin, Vladimir Vinokurov

**Affiliations:** 1 Department of Physical and Colloid Chemistry, National University of Oil and Gas “Gubkin University”, 119991 Moscow, Russiacherednichenko.k@gubkin.ru (K.A.C.); semenov.a@gubkin.ru (A.P.S.); vladimir@vinokurov.me (V.V.); 2 Institute of Fundamental Medicine and Biology, Kazan Federal University, Kreml uramı 18, 42000 Kazan, Republic of Tatarstan, Russia; kazanbio@gmail.com; 3 Department of Chemistry, Stephenson Institute for Renewable Energy, University of Liverpool, Liverpool L69 7ZD, UK; shchukin@liverpool.ac.uk

**Keywords:** phase-change materials, encapsulation, interfacial polymerization, polyisocyanate, elastic polyurethane, thermoregulating paint

## Abstract

Organic phase-change materials (PCMs) hold promise in developing advanced thermoregulation and responsive energy systems owing to their high latent heat capacity and thermal reliability. However, organic PCMs are prone to leakages in the liquid state and, thus, are hardly applicable in their pristine form. Herein, we encapsulated organic PCM *n*-Octadecane into polyurethane capsules via polymerization of commercially available polymethylene polyphenylene isocyanate and polyethylene glycol at the interface oil-in-water emulsion and studied how various *n*-Octadecane feeding affected the shell formation, capsule structure, and latent heat storage properties. The successful shell polymerization and encapsulation of *n*-Octadecane dissolved in the oil core was verified by confocal microscopy and Fourier-transform infrared spectroscopy. The mean capsule size varied from 9.4 to 16.7 µm while the shell was found to reduce in thickness from 460 to 220 nm as the *n*-Octadecane feeding increased. Conversely, the latent heat storage capacity increased from 50 to 132 J/g corresponding to the growth in actual *n*-Octadecane content from 25% to 67% as revealed by differential scanning calorimetry. The actual *n*-Octadecane content increased non-linearly along with the *n*-Octadecane feeding and reached a plateau at 66–67% corresponded to 3.44–3.69 core-to-monomer ratio. Finally, the capsules with the reasonable combination of structural and thermal properties were evaluated as a thermoregulating additive to a commercially available paint.

## 1. Introduction

According to the International Energy Agency (IEA), heat generation constitutes approximately 50% of consumed primary energy and contributes 40% of carbon dioxide emission [1]. Among this, about 46% of the generated heat is consumed in buildings, including a half of this energy, which is used for indoor and water heating. Despite the developments in renewable energy, fossil fuels keep dominating heat supplies, covering over 60% of heating energy demand [2]. The consumption of fossil-fuel-based heating in buildings can be mitigated by responsive energy management employing smart thermoregulating materials [3,4]. The concept of smart thermal regulation implies the storage of the accessible thermal energy and its on-demand release in response to environmental changes [5]. Responsive energy management is among the prerequisites in developing zero-energy thermoregulation systems that will simultaneously reduce thermal energy requirements in buildings and improve indoor comfort [6,7].

Responsive thermal regulation can be achieved through the storage and release of latent heat during the melting and crystallization in phase-change materials (PCMs) [8]. If compared to conventional sensible heat storage systems, latent heat storage is more reliable owing to higher energy density storage in the narrow temperature range and absence of heat leakages at any temperature [9]. Among others, certain organic PCMs, e.g., paraffins and fatty acids, possess high melting and freezing enthalpies, a wide range of phase transition temperatures defined by the length of the methylene chain, and durability of thermal properties [10,11]. However, the applicability of bare PCMs is limited, as they are prone to leakages during the solid–liquid transition. Shape stability can be improved by the deposition of PCMs on substrates with enhanced surface area or by their encapsulation into micro- and nanocontainers [12,13,14]. The encapsulation seems preferable as the restriction of PCMs into impermeable micro- and nanoshells foremost prevents their leakage and environmental exposure as well as improves thermal reliability and increases the heat transfer area comparing to the bulk form [15,16].

Emulsion interfacial polymerization is regarded as a versatile and feasible way to prepare micro- and nanocapsules or porous templates [17,18,19,20,21]. This method is based on the formation of direct (oil-in-water) or inverse (water-in-oil) emulsions containing different monomers dissolved in each phase, which are able to react at the oil/water interface of the emulsion droplet. Ordinary, the polymerization is triggered by pH or temperature adjustment. For encapsulation, the target agent must be dissolved in the core phase that will be enclosed into a polymeric shell, as the reaction is complete. The combination of nanoscale building blocks arranged via self-assembly into microscale capsules further embedded within the macroscale surface coating falls within the paradigm of nanoarchitectonics [22].

The reaction between isocyanates and polyols holds promise as a method for encapsulation of PCMs via polymerization of polyurethane (PU) shell at the oil/water interface. The most commonly used isocyanates are either aromatic or aliphatic compounds, and thus can be naturally included into the oil phase, while several polyols are soluble in water. Cyanate and hydroxyl groups give rise to urethane linkages referred as “hard domains” in PU structure, owing to the intergroup association by hydrogen bonds. The hard domains are interconnected with mobile polymeric chains of polyols, which represent the “soft domains” [23]. As a result, the PU shell combines the strength and elasticity, the essential prerequisites for reliable encapsulation of materials intended to undergo the reversible phase transitions. Additionally, PUs are stable in water and common organic solvents and, therefore, PCM-loaded PU capsules are highly promising as a thermoregulating additive to various conventional construction materials, e.g., dry building mixes, finishing layers, and paints [24].

The preparation of energy capsules with encapsulated organic PCMs via interfacial polymerization was reported previously [25,26,27,28,29]. Cho et al. described the capsules loaded with octadecane and polyurea shell formed by the polycondensation of toluene-2,4-diisocyanate (TDI) and diethylenetriamine (DETA) and found that encapsulation efficiency of paraffin depends on the ratio of oil to water phase [25]. Salaün et al. studied the formation of polyurea–urethane microparticles containing xylitol through a reaction between methylene diphenyl diisocyanate (MDI) and xylitol [26]. Therein, the xylitol was used as a monomer, simultaneously forming the shell and the PCM. The reported encapsulation efficacy was 77% with the latent heat of fusion of 196 J/g, whereas the core to shell ratio was 77:23 wt%. Lu et al. described the formation of double-shell capsules containing butyl stearate [27]. The outer capsule shell was polyurea formed through polycondensation of TDI and DETA, and the inner shell was polyurethane formed through polycondensation of TDI and polypropylene glycol 2000. The capsules had latent heat capacity of 85 J/g with the loading efficiency of 95%. Yoo et al. reported the synthesis of the capsules with poly(urea–urethane) shell reinforced with cellulose nanocrystals and loaded with methyl laurate [28]. At their best, the resulted capsules had the latent heat of fusion of 148 J/g corresponding to the loading efficiency of 66%. The loading efficiency and shell thickness of the microcapsules increased as the core/shell weight ratio decreased. Cai et al. described a preparation of polyurea capsules via co-solvent free interfacial polycondensation of TDI and DETA and dodecanol dodecanoate as core material [29]. The capsules demonstrated the latent heat storage capacity of 139 J/g with the encapsulation efficiency of the PCM of 74%.

De Castro et al. reported the fabrication of polyurethane capsules loaded with *n*-Docosane prepared via interfacial polymerization of MDI and PEG 1000 [30,31]. In these studies, the authors described the synthesis procedure and studied the thermal properties and confinement effect on the phase transition of encapsulated PCM. However, despite the employed *n*-Docosane having a high enthalpy of phase transitions of 248 J/g, the reported capsules demonstrated relatively low latent heat storage performance varying from 48 to 88 J/g corresponding to the actual content of *n*-Docosane of only 19–32% depending on the capsule size. An important limitation of these works needs to be noted, that is the authors studied the PU capsules synthesized with the only composition of the oil phase containing the same amount of PCM and did not investigate the effects of the variation in mass fraction of paraffin in the oil core affecting the structure and latent heat storage performance of the resulted capsules.

Herein, we demonstrate the preparation of PU capsules with a various loading of paraffin PCM *n*-Octadecane and studied how the increase in paraffin content in the initial oil phase affected the formation of the PU shell, structure, and latent heat storage capacity of the resulted capsules. Unlike the previous publications, we did not vary the ratio of the oil to water phase and the ratio of PU monomers dissolved in them. What was of additional interest is the possibility to prepare the PU capsules with commercially available and cheap polymethylene polyphenylene isocyanate (PAPI) instead of more commonly employed laboratory grade MDI and TDI. The resulting capsules were intact and unaggregated, although polydisperse with the mean size from 9.4 to 16.7 µm. We demonstrated that at the same volume of the solvent and the same monomer amount, the increase in *n*-Octadecane feeding during the emulsion preparation resulted in an increase in its actual encapsulated content; however, this led to a reduction in thickness of the PU shell. Finally, we have evaluated the effect of the prepared *n*-Octadecane-loaded capsules with the best structural and thermal properties on the thermoregulating performance of the commercially available acrylic paint.

## 2. Materials and Methods

### 2.1. Materials

Polymethylene polyphenylene isocyanate Wannate PM-200 (PAPI) with an average functionality of 2.6–2.7 cyanate groups per molecule was purchased via a local vendor from Yantai Wanhua Polyurethanes (Yantai, China). *n*-Octadecane (technical grade 90%) was obtained from Alfa Aesar (Kandel, Germany). Poly(ethylene glycol) with molecular weight of 1000 g/mol (PEG 1000), 4,N,N-Trimethylaniline (TMA, 99%), Poly(vinyl alcohol) (PVA, 99+% hydrolyzed), and toluene (≥99.5%) were purchased from Sigma-Aldrich (Darmstadt, Germany).

Deionized (DI) water with the specific resistivity no lower than 18.2 MΩ·cm was used in all steps of synthesis involving the preparation of aqueous solutions and rinsing.

### 2.2. Synthesis of n-Octadecane-Loaded Polyurethane Capsules

The encapsulation of *n*-Octadecane was carried out with the emulsion interfacial polymerization technique. In particular, the oil phase included 18 mL of toluene, 3.2 g of PAPI, and various portions of *n*-Octadecane. The feeding of *n*-Octadecane was 1.6, 3.2, 4.8, 6.4, and 8 g. The aqueous phase was 80 mL of 2 wt% PVA solution. The PVA solution was mixed with the oil phase and stirred with a magnetic stirrer at 600 rpm to obtain an initial macroemulsion. The mixture was further homogenized with T 18 Ultra-Turrax disperser (IKA) at 15,000 rpm for 5 min to obtain the microemulsion. Further, the shell polymerization was carried out in a three-neck round-bottomed flask equipped with a reflux condenser and a thermometer. Upon the emulsion being heated to 70 °C under a gentle stirring, 3.2 g of PEG 1000 and 300 µL of TMA were added. The stirring was maintained for additional 2 h at 70 °C to let the reaction flow and complete the PU shell formation. As the polymerization was completed, the capsules were rinsed with DI water and separated from the liquid phase by centrifugation (4000 rcf, 10 min). The collected slurry was dried at ambient temperature in the desiccator, yielding a white to light-yellow powder. The capsule samples referred as PU-1, PU-2, PU-3, PU-4, and PU-5 with the number index increased along with the *n*-Octadecane feeding to the oil phase.

### 2.3. Characterization of the PU Capsules

The morphology and shell thickness of the prepared capsules were studied with scanning electron microscopy (SEM) employing an SEM-FIB JIB-4501 (JEOL, Akishima/Tokyo, Japan) microscope. The capsule suspensions were dropped onto the tables covered with a conductive carbon tape, dried, and sputtered with a thin gold layer. The SEM images were captured at an accelerating voltage of 5 kV. To evaluate an average thickness of capsule shells, at least 15 images of each sample were taken and processed with ImageJ software.

Additionally, the capsule structure was studied with a transmission electron microscopy (TEM). The images were acquired with a JEM 2100 UHR LaB_6_ (JEOL, Akishima/Tokyo, Japan) microscope equipped with Quemesa (Olympus, Shinjuku/Tokyo, Japan) 11 MegaPixel CCD camera. The accelerating voltage was 200 kV. The drops of capsule suspensions were deposited onto Lacey formvar/carbon grids (Ted Pella, Inc., Redding, CA, USA). In order to remove organic moieties, the grids were treated with Heinniker Plasma HPT-100 cleaner before the measurements were performed.

The successful polymerization of polyurethane at the oil/water interface of emulsion droplets and trapping of *n*-Octadecane within the PU shell were confirmed with Fourier transformed infrared spectroscopy (FTIR). The absorbance spectra were acquired with a Nicolet iS10 (Thermo Scientific, Waltham, MA, USA) FTIR spectrometer with germanium ATR crystal in the 4000–600 cm^−1^ range. The final spectra were derived from 16 serial scans.

Additionally, the formation of the PU shell was visualized using an Eclipse Ni-E A1 (Nikon, Tokyo, Japan) laser scanning confocal microscope. The samples were collected at different stages of capsule preparation: after preparation of the PVA-stabilized emulsion, after addition of PEG 1000 and TMA when the shell crosslinking was initialized, and after completion the formation of PU shell. The images were acquired in the transmittance mode.

The capsule size distribution was measured using a Master Sizer 3000 laser diffractometer (Malvern Panalytical, Malvern, UK). Laser diffractometry operates with volume-weighted size distribution; therefore, a mean capsule size was determined according to the measured volume-weighted mean diameter. The mean diameter distribution was evaluated by Span, calculated as follows:

(1) $$\mathrm{Span}=\frac{\mathrm{Dv}90-\mathrm{Dv}10}{\mathrm{Dv}50},$$

where Dv90 is the diameter belonging to 90% of the sample, Dv10 is the diameter belonging to 10% of the sample, and Dv50 is the median volume-weighted diameter in the distribution. The mean dimeter distribution was evaluated employing an analysis model for non-spherical capsules.

The latent heat capacity and thermal reliability of the encapsulated *n*-Octadecane were measured using a DSC 214 Polyma (Netzsch, Selb, Germany) calorimeter in the temperature range from −20 to 70 °C with a 10 °C/min ramp. The sample mass was 5 mg. The collected melting and crystallization curves were further processed to subtract the baselines. Finally, time integration of exothermic and endothermic peaks was performed to determine the enthalpy of phase transitions of the loaded *n*-Octadecane with commonly employed data handling software.

The leakage rate of *n*-Octadecane was evaluated by incubation of the dried PU capsules in hexadecane, which is a good solvent for paraffins. To do this, approximately 100 mg of the capsules was incubated in 2 mL of After that, the capsules were separated by centrifugation and dried at ambient temperature until the mass levelled off. The leakage rate was figured out with respect of the capsule mass loss (*LR_m_*) and reduction in actual *n*-Octadecane content according to DSC (*LR_E_*).

The thermal decomposition patterns of the PU capsules were established with thermal gravimetry analysis (TGA) employing an STA 449 F5 Jupiter (Netzsch, Selb, Germany) device. The temperature range was 30–800 °C. The measurements were carried out at a ramp of 10 °C/min under the nitrogen atmosphere. The sample mass was 6 mg.

### 2.4. Preparation of the Thermoregulating Material Containing PU Capsules

Thermoregulating coating was prepared by addition of concentrated aqueous suspension of PU capsules to commercially available acrylic paint. The structure of the resulted composite as well as the distribution and integrity of the PU capsules in the hardened paint layer were studied with SEM. The effect of the PU capsules on the latent heat capacity and reliability of the thermoregulating properties was studied with DSC. Finally, the effect of the PU capsules on the cooling rate of the paint layer was evaluated by thermal imaging. The thermal images were acquired with the Guide D400 infrared camera (Guide Sensmart, Wuhan, China). To achieve this, the paint with PU capsules was brushed onto steel plates. The plates were heated in the drying chamber at 60 °C for 25 min and then were placed into a refrigerator at 3 °C for 25 min to measure the cooling rate of the paint layer with an IR camera. The measurements were carried out 3 times. The collected thermal data were processed with ThermoTools (v 3.3.3.0_QDZG, Guide Sensmart, Wuhan, China) software to plot averaged cooling curves. The paint coating without the addition of PU capsules was employed as a reference.

## 3. Results and Discussion

### 3.1. PU Capsule Synthesis and Characterization

**Scheme 1:**
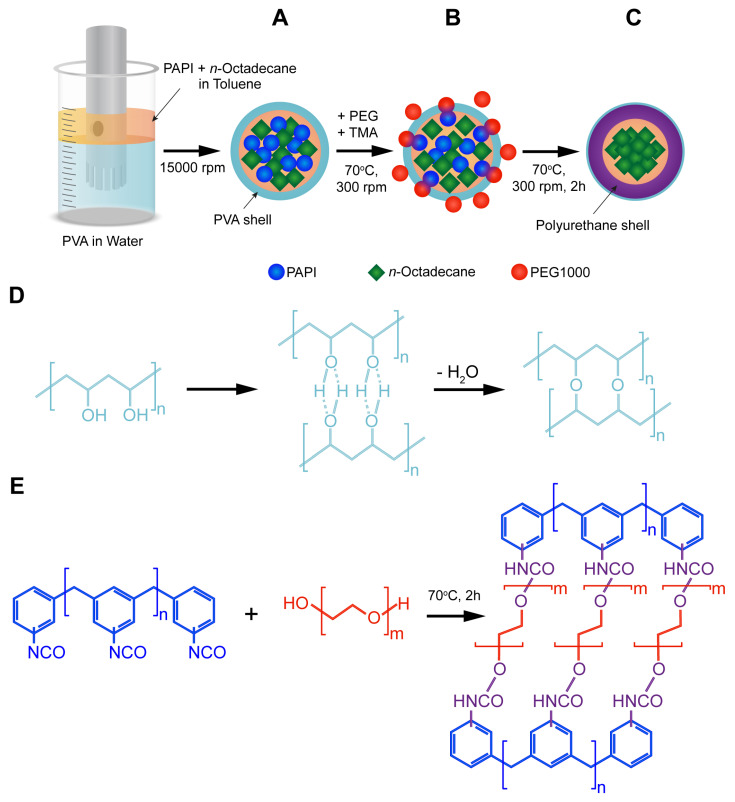
Preparation of the polyurethane capsules loaded with *n*-Octadecane via interfacial polymerization of PAPI and PEG 1000: (**A**) preparation of PVA-stabilized oil-in-water emulsion containing PAPI and *n*-Octadecane dissolved in the oil phase; (**B**) initiation of polymerization through the reaction between PAPI and PEG at the oil/water interface; (**C**) polymerization of the polyurethane shell and entrapment of *n*-Octadecane within the capsule interior; (**D**) possible mechanism of formation of PVA surface layer; (**E**) possible mechanism of reaction between PAPI and PEG and resulted polyurethane structure.

The synthesis of the *n*-Octadecane-loaded microcapsules with a PU shell was performed via emulsion interfacial polymerization technique. Scheme 1 shows a step-by-step preparation procedure. The first step involved the creating of PVA-stabilized oil-in-water emulsion with the oil phase consisting of PAPI and *n*-Octadecane dissolved in toluene. PVA has a number of advantages as an emulsifier. First, PVA tends to stabilize oil drops in aqueous medium owing to its amphiphilic nature, that is, the vinyl monomers interact with the oil phase while the hydroxyl groups are oriented towards the water phase [32]. Additionally, PVA is capable to form an interconnected network at an oil/water interface via hydrogen bonding and further possible chemical crosslinking of hydroxyl groups (Scheme 1A) [33,34]. Further, PEG 1000 and TMA were introduced, and the mixture temperature was increased to 70 °C to start the polymerization (Scheme 1B). TMA is a tertiary amine with low steric hindrance that is typically employed to catalyse a polyurethane formation in reaction between isocyanates and alcohols [35]. The commercially available PAPI is a mixture that mainly consists of 4,4′-methylenediphenyl diisocyanate with inclusion of 2,4′-methylenediphenyl diisocyanate, 2,2′-methylenediphenyl diisocyanate, and higher homologues. Therefore, heating is a necessary condition to eliminate a steric factor and improve the reactivity of isocyanate groups in various MDI isomers [23]. Furthermore, the formation of the PU shell is driven by the moving boundary mechanism. This implies the diffusion of polyol to the reaction site at the oil/water interface through the initially formed polymeric membrane layer [36]. Thus, the rise in reaction temperature in its turn promotes the polyol diffusion. It should be noted that formation of urethane is attended by the heat release [ΔH = 24 Kcal/mol] [23]. Therefore, the successful polymerization of the capsule shell can be recognized by the further rise in the temperature of reaction mixture beyond 70 °C. The reverse cooling of the mixture to 70 °C indicated the completion of the polymerization. A possible way of formation of the crosslinked polyurethane shell through the polymerization of PAPI and PEG 1000 in shown in Scheme 1C.

**Figure 1:**
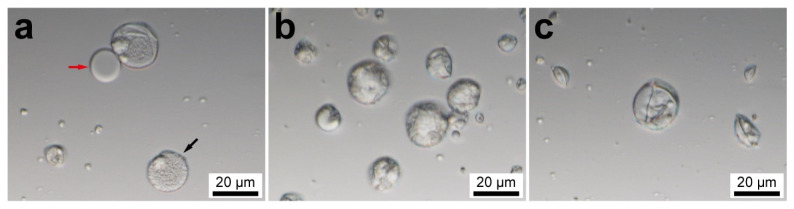
Confocal microscopy images of the PU capsules at different reaction stages: (**a**) as-prepared PVA emulsion (the red arrow shows the liquid content released due to disruption of PVA layer, the black arrow shows an intact emulsion droplet); (**b**) at the beginning of the shell polymerization after addition of PEG and TMA; (**c**) PU capsules after completion of the reaction.

The preparation of the PU capsules at different reaction stages was imaged using confocal microscopy. Figure 1a shows the optical transmittance image of PVA emulsion droplets obtained in the first preparation step. The PVA layer is thin and soft; as a result, the droplets can be easily broken. The liquid core (marked by a red arrow) is clearly distinguished from the polymeric shell. This confirms the successful encapsulation of the oil phase. Figure 1b shows the capsules at the intermediate stage of the PU shell formation when the reaction between PEG and PAPI is incomplete and the temperature of the reaction mixture is maintained above 70 °C. The capsules appear opaque and tighter due to initialization of PEG and PAPI crosslinking. As the reaction time increases, a robust PU shell is formed due to completion of the crosslinking (Figure 1c). Some capsules appear shrunken and curved inward, which can be related to the deformation of the oil core during the mixing.

**Figure 2:**
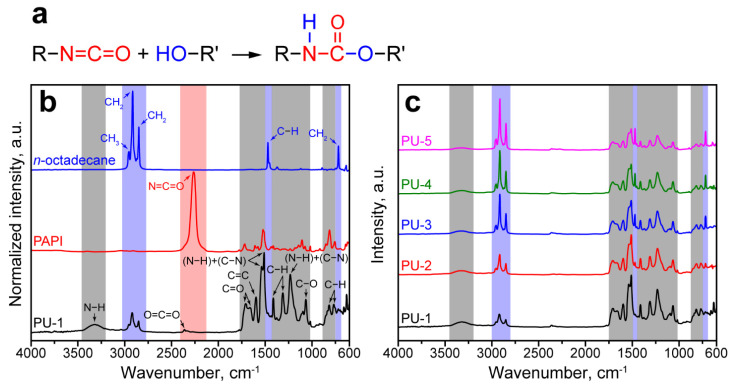
(**a**) The scheme of reaction between isocyanate and alcohol; (**b**) FTIR spectra of *n*-Octadecane, PAPI, and PU-1 capsules (blue, red, and grey backgrounds mark the bands of the corresponding functional groups); (**c**) comparison of FTIR spectra of PU capsules with various *n*-Octadecane loading.

The successful polymerization of the PU shell and encapsulation of paraffin were confirmed with FTIR spectroscopy. As seen in Figure 2a, the formation of polyurethanes in a common reaction between isocyanates and alcohols involves the addition of a hydrogen atom at the N=C bond in the isocyanate −N=C=O group [23]. The resulted urethane −NHCOO− group can be identified though the newly absorption maximums owing to vibrations of N−H and C−N bonds and the absence of absorbance due to asymmetric stretching of the isocyanate groups at 2265 cm^−1^ [37].

Figure 2b demonstrates the FTIR absorbance spectra of bare *n*-Octadecane, PAPI, and PU-1 capsules. The FTIR spectra of paraffin and PAPI revealed the intrinsic absorption bands due to the stretching and bending of their functional groups.

The spectrum of PU-1 capsules exhibited the inherent polyurethane peaks with absorption maximums of N−H, C=O, and C−N bands, which were not detected in the paraffin and PAPI spectra. In particular, the peak at 3305 cm^−1^ corresponded to the stretching vibration of N−H bond. In addition, several absorption maximums were detected in the 1800–1000 cm^−1^ band. The peak at 1730 cm^−1^ corresponded to the stretching vibration of the C=O group; the peak at 1600 cm^−1^ belonged to vibrations of C=C in the benzene ring; the double peak at 1538–1512 cm^−1^ comes from vibration of hydrogen-bonded and free (N−H)+(C−N) groups; the peaks at 1410 and 1310 cm^−1^ corresponded to asymmetric bending stretching and symmetric bending vibration of C−H group; the peak at 1230 cm^−1^ belonged to bending vibration of N−H and stretching vibration of C−N groups; the peak at 1065 cm^−1^ was due to symmetric stretching vibration of C−O−C; two peaks at 814 cm^−1^ and 765 cm^−1^ came from out-of-plane bending vibration of C−H [38]. The spectrum of PU-1 capsules had no absorption peak at 2265 cm^−1^ due to the −N=C=O group [37]. This confirmed that all PAPI successfully reacted with PEG. Additionally, the peaks at 2361 cm^−1^ and 1662 cm^−1^ corresponding to the stretching vibration of O=C=O and C=O groups were detected. This suggests that PAPI may also react with water with the generation of CO_2_. Further, CO_2_ participated in the formation of O=C=O and distributed urea −NHCONH− groups that resulted in a shifted to 1662 cm^−1^ absorption of C=O group [37,39].

Furthermore, the absorption maximums corresponded to symmetric and antisymmetric stretching of CH_2_ groups at 2915 and 2849 cm^−1^ and antisymmetric stretching of the CH_3_ group at 2954 cm^−1^ were detected in the spectrum of PU-1 capsules [40]. This confirmed the successful encapsulation of *n*-Octadecane. Figure 2c shows the comparison of FTIR spectra of the microcapsules prepared with various *n*-Octadecane feeding. The increase in paraffin content affects the intensity of paraffin peaks if compared with the peaks corresponding to PU shell. Starting from PU-2 capsules, the peaks at 1470 and 717 cm^−1^ from the stretching vibration of the C−H group and the rocking vibration of the CH_2_ group of the paraffin aliphatic chain, respectively, can be clearly distinguished.

**Figure 3:**
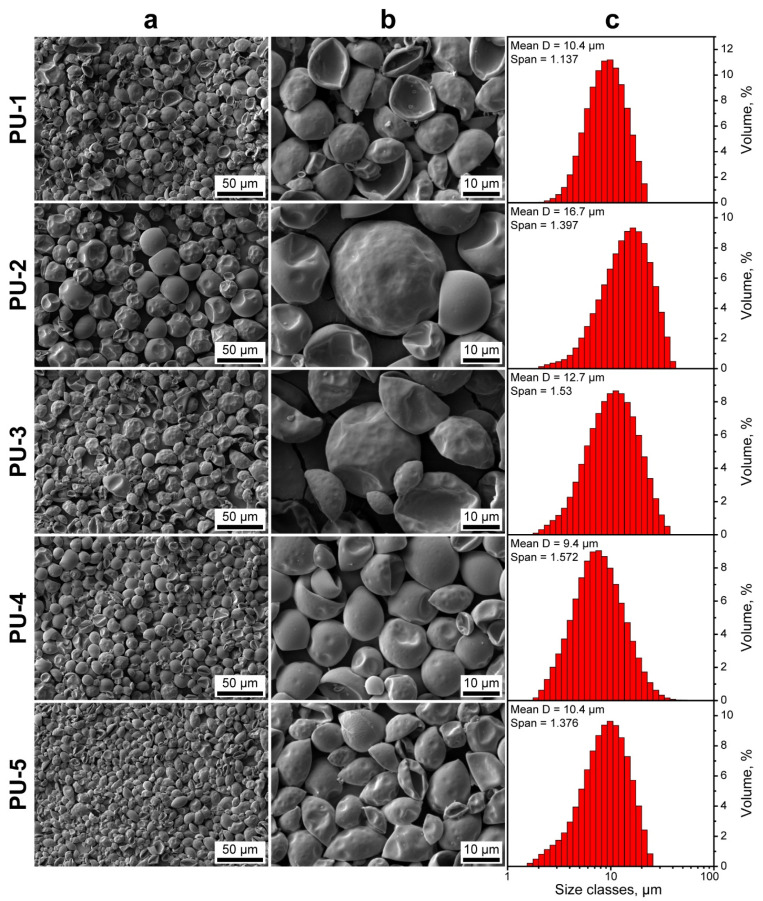
(**a**,**b**) SEM images of the PU capsules with various *n*-Octadecane feeding at various magnifications; (**c**) size distribution of PU capsules measured with laser diffractometry.

Figure 3a,b shows the electron microscopy images of the obtained PU capsules. The capsules had a close to spherical shape with uniform and smooth surface. The smooth surface morphology of capsules can be derived from the PVA stabilizing layer. PVA tends to form an organized and ordered structure at the oil/water interface owing to its linear nature. This facilitates the homogeneous diffusion of PEG 1000 and, thus, uniform polymerization over the droplet surface [41,42]. Additionally, the SEM images suggest that the capsules had an elastic shell. This is typical to reactions of isocyanates with linear polyether polyols of low functionality with 2–3 terminal hydroxyl groups per molecule, like PEG [23]. The long linear polyol chain of PEG 1000 promotes the formation of soft domains in the polyurethane structure and reduces the association of hard domains of urethane groups via hydrogen bonding [43]. The PU-1 capsules are mostly curved inward apparently due to the low content of stiff fraction of encapsulated *n*-Octadecane in their interior. The increase in *n*-Octadecane feeding led to more spherical capsules, as evidenced by PU-2, PU-3, and PU-4 samples. However, PU-5 capsules with the highest intended *n*-Octadecane content also had a mostly curved shape. This can be related to the reduction in PAPI solubility in toluene as the additional *n*-Octadecane was added to the oil phase [27]. As a result, PAPI segregated from the oil phase and, thus, less isocyanate was available to react with PEG at the emulsion interface. Eventually, this led to a thinner and less crosslinked capsule shell. The segregation of PAPI from the oil phase was indirectly confirmed by the sedimentation of solid polyurethane particles in the reaction mixture. Unlike the solid PU particles, the PU capsules with *n*-Octadecane core tended to float to the upper layers of the aqueous medium and can be easily separated by centrifugation. The laser diffractometry revealed the monomodal capsule size distribution with the mean diameter in the range from 9.4 to 16.7 µm. The span values varied from 1.14 to 1.58, indicating relatively narrow capsule size distribution.

**Figure 4:**
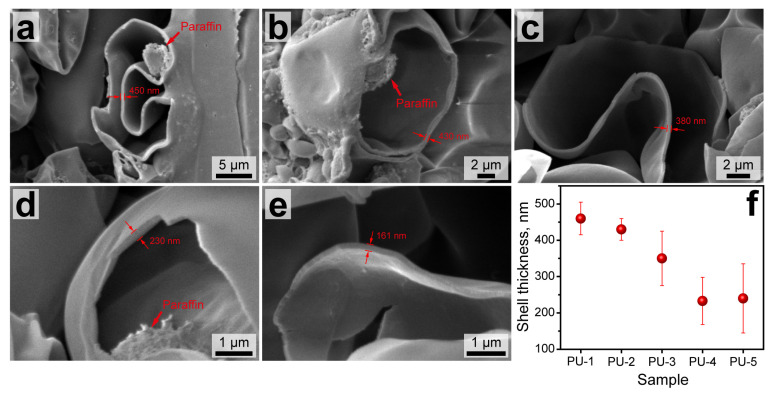
SEM images of the sliced PU-1 (**a**), PU-2 (**b**), PU-3 (**c**), PU-4 (**d**), and PU-5 (**e**) capsules showing the cross section of their shells; (**f**) dependence of the average shell thickness of PU capsules on the content of the loaded *n*-Octadecane figured out from the SEM images.

The structure of the capsule shells was also studied with SEM. To achieve this, drops of capsule suspensions were dried and sliced with a sharp lancet. Then, the samples were placed on the tables in the way the drop cross sections are normally oriented to the microscope detector. Figure 4a–e shows the representative SEM images of the capsule cross-sections. The capsules prepared with a low *n*-Octadecane feeding (PU-1 and PU-2) had a relatively thick shell with an average thickness of 460 to 430 nm, which corresponded to the previously reported PU capsules with a comparable paraffin loading [30,31]. However, the further increase in paraffin content in the oil phase was attended with a gradual reduction in PU shell thickness. Although the shell thickness may vary depending on the capsule size, an averaged value for PU-3 capsules was worked out to be about 350 nm and 220–230 nm for the PU-4 and PU-5 capsules, respectively (Figure 4f). Additionally, the higher magnification images in Figure 4d,e reveal a single layer shell structure, which suggests an interpenetrating polymeric network between PVA and PU layers was formed. Apparently, PAPI monomers reacted with PEG on the border of the PVA layer, forming an initial polyurethane phase that was further developed to the PU shell according to the moving boundary mechanism [32].

**Figure 5:**
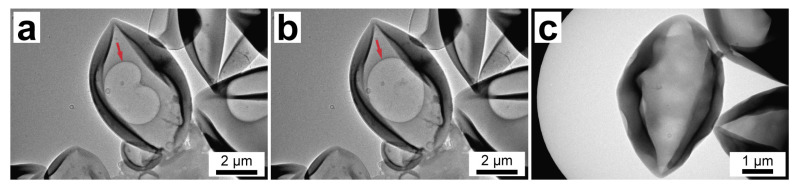
(**a**,**b**) TEM images of PU-2 capsules showing the melting of *n*-octadecane in capsule interior under exposure to electronic beam (the red arrow indicates an air bubble); (**c**) TEM image of PU-3 capsules.

To take a closer look at the capsule structure, TEM studies were performed. Figure 5 shows the TEM images of PU-2 (Figure 5a,b) and PU-3 capsules (Figure 5c). The images of PU-2 capsules clearly demonstrate there is an air bubble in the capsule interior, which can be recognized as a more transparent area inside the capsule (designated with an arrow). Furthermore, the shape of the air bubble changes under electronic beam exposure owing to the melting of paraffin within the capsule that confirms its reliable loading and durability of the formed PU shell (the video of the paraffin melting available online as a Supplementary Material Video S1). On the other hand, PU-3 capsules of the comparable size demonstrate more uniform interior without any bubbles inside, indicating higher capsule loading with a solid paraffin fraction.

### 3.2. Latent Heat Storage Performance and Thermal Stability

The study of the latent heat capacity of the PU capsules prepared with various *n*-Octadecane feeding was carried out with DSC. The melting curve of the bare *n*-Octadecane demonstrated a single endothermic peak with a melting point at 30 °C and enthalpy Δ*H_M_* = 197 J/g [44]. The crystallization curve of *n*-Octadecane revealed a single exothermic peak with the crystallization point of 17 °C, yet two prominent segments in the cooling curve can be distinguished. The first one is related to the heterogeneous nucleation of *n*-Octadecane and formation of *α*-form or the rotator (R_I_) phase [45]. The second segment is defined by the homogeneous nucleation and formation of *β*-form or the triclinic crystalline phase of *n*-Octadecane [46,47]. The overall latent heat released in these two stages (Δ*H_C_*) was 196 J/g.

**Figure 6:**
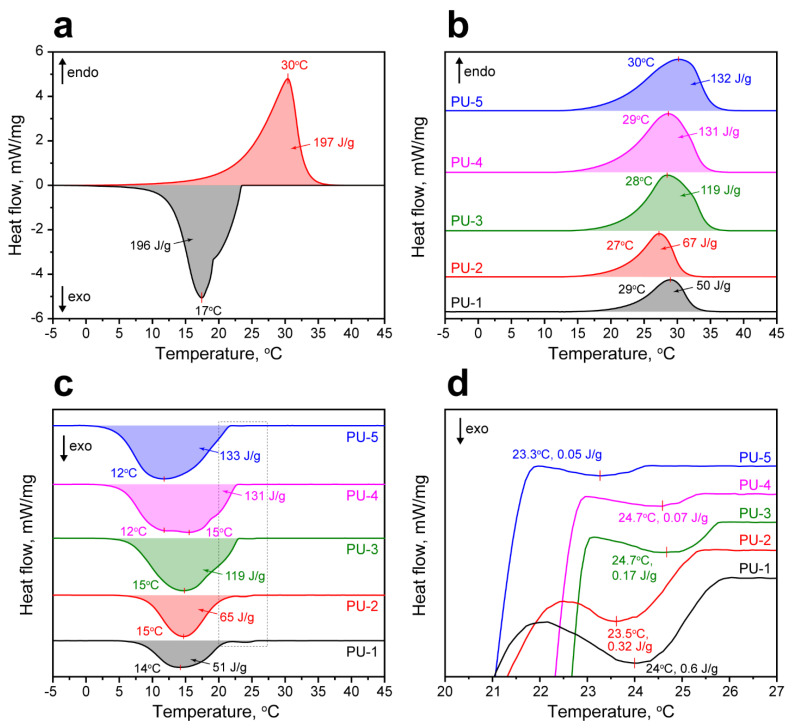
(**a**) Melting and crystallization curves of the bare *n*-Octadecane; (**b**) melting curves of the PU capsules; (**c**) crystallization curves of the PU capsules; (**d**) exothermic peaks on crystallization curves of the PU capsules indicating the surface freezing of the microencapsulated *n*-Octadecane.

The melting point of the encapsulated *n*-Octadecane slightly varied in the range from 27 to 30 °C (Figure 6b). Predictably, the melting enthalpy of the microcapsules had reduced proportional to the loaded amount of PCM and varied from 50 to 132 J/g along with *n*-Octadecane feeding. The crystallization points of the encapsulated *n*-Octadecane varied from 12 to 15 °C (Figure 6c). The crystallization enthalpy also had reduced with respect to the bare *n*-Octadecane and was found to be 51–133 J/g depending on the added PCM. Noteworthy, the crystallization behaviour of the bare and microencapsulated *n*-Octadecane was somewhat different. Foremost, the crystallization curves of encapsulated *n*-Octadecane clearly demonstrated small exothermic peaks at 23.3–24.7 °C due to the surface freezing phenomenon (Figure 6d). The surface freezing is the formation of the crystalline monolayer on the surface of long-chained (from C_15_ and higher) *n*-alkanes when the temperature exceeds the crystallization point [48]. Due to the larger surface-to-volume ratio of the encapsulated *n*-Octadecane compared to the bulk, the surface freezing had a more discernible effect on its crystallization. The enthalpy of surface freezing was 0.6–0.05 J/g and reduced as paraffin feeding increased [45]. This suggests that at low *n*-Octadecane feeding, the smaller paraffin cores are formed and, therefore, the surface freezing is more prominent. As the *n*-Octadecane feeding increased, the core size also increased and the impact of the surface freezing appeared less significant. This is in agreement with the TEM results showing the various occupations of the PU capsule interior. Additionally, the encapsulated *n*-Octadecane demonstrated the shift of crystallization point towards lower temperatures to 2–5 °C. This can be associated with the reduced number of the nucleating seeds that resulted in delayed nucleation compared with the bulk paraffin [49].

To further investigate the relationship between structural and latent heat storage properties in PU capsules, the core-to-monomer ratio and PCM-to-toluene ratio were calculated. To do this, an actual *n*-Octadecane core content regarding the measured melting and freezing enthalpies was calculated as follows:

(2) $$E_{a}=\frac{\Delta H_{M/caps}+\Delta H_{F/caps}}{\Delta H_{M/PCM}+\Delta H_{F/PCM}}\times 100\%,$$

where Δ*H_M/caps_* and Δ*H_F/caps_* are the melting and freezing enthalpies of the encapsulated PCM while Δ*H_M/PCM_* and Δ*H_F/PCM_* are the melting and freezing enthalpies of the initial PCM.

The theoretical *n*-Octadecane core content in the PU capsules is expressed as follows:

(3) $$E_{t}=\frac{m_{PCM}}{m_{PCM}+m_{M}}\times 100\%,$$

where *m_PCM_* is the mass of the initially added *n*-Octadecane, and *m_M_* is the mass of the monomers (PAPI and PEG) employed for PU shell formation.

The core-to-monomer ratio was calculated as follows:

(4) $$CM=\frac{m_{PCM}+m_{S}}{m_{M}}\times 100\%,$$

where *m_PCM_* is the mass of *n*-Octadecane, *m_S_* is the mass of the solvent (toluene), and *m_M_* is the mass of the monomers.

The PCM-to-toluene ratio was calculated as the volume ratio of *n*-Octadecane to toluene. The PU capsule composition and the calculated ratios are listed in Table 1.

**Figure 7:**
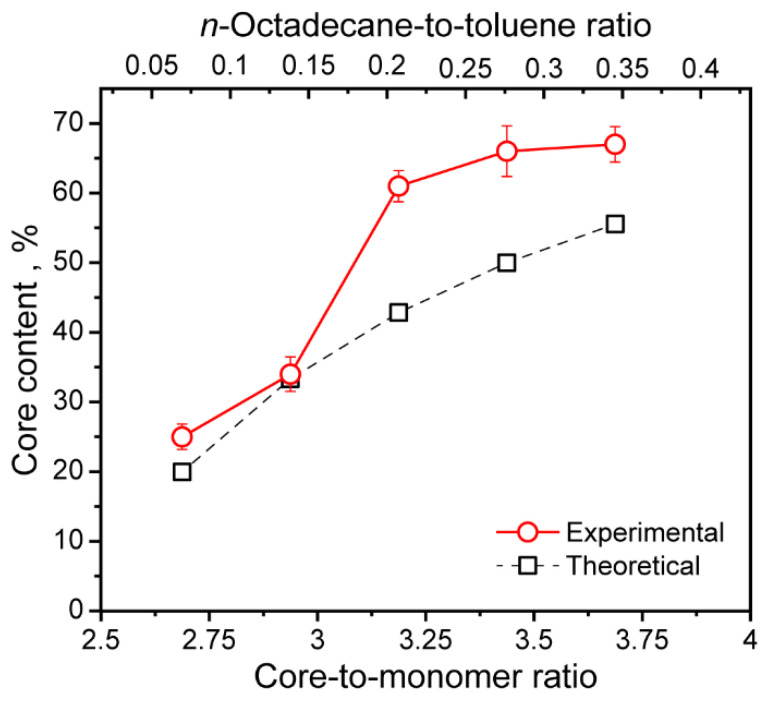
Dependence of experimentally measured (actual) and theoretically calculated *n*-Octadecane core content in the PU capsules on the core-to-monomer ratio and *n*-Octadecane-to-toluene ratio.

The dependence of the measured and theoretically calculated *n*-Octadecane content on the core-to-monomer ratio is shown in Figure 7. Noteworthy, the most prominent growth of the actual *n*-Octadecane core content from 25 to 61% was found when the core-to-monomer ratio increased from 2.69 to 3.19. This corresponds to the increase in the *n*-Octadecane feeding from 1.6 to 4.8 g. The further increase in paraffin feeding resulted only in an insufficient improvement in the actual core content, and therefore, in the latent heat storage and release performance. Starting from 6.4 g of the added *n*-Octadecane, the actual core content levelled off at around 66%. The highest actual core content of 67% was reached at 8 g of the added paraffin, which corresponded to the core-to-monomer ratio of 3.69. According to previous studies, the core-to-monomer ratio of 3–4 was found as an optimal to form a stable polymeric shell around the PCM core [27,50]. Furthermore, comparing to the previously reported paraffin-loaded PU microcapsules [30,31], PU-3, PU-4, and PU-5 capsules demonstrated twice as much increase in actual PCM content according to DSC.

Importantly, the experimentally measured core content was higher than the theoretical one. Taking into account that the mass of the monomers is in the denominator in Equation (3), the *m_M_* value should be reduced in order the experimentally measured *n*-Octadecane content meets the theoretical one. This suggests that not all the added PAPI and PEG were involved in the capsule shell polymerization, as was evidenced by PAPI segregation from the oil phase.

Figure 7 also shows the dependence of the actual core content on the *n*-Octadecane-to-toluene ratio. The ratio varied from 0.07 to 0.35, which is relatively low. This explains the curved shape of the resulted PU capsules as a low PCM-to-solvent ratio gave rise to the less viscous capsule core. The fluid core can easily undergo shear deformation during the stirring [50]. On the other hand, the fluid core is preferable in interfacial polymerization to promote the diffusion of the monomer dissolved in the oil phase.

Finally, the efficiency of accumulation and release of latent heat during the melting and crystallization of *n*-Octadecane restricted within the capsule cavity can be evaluated with thermal storage capability *η* as follows:

(5) $$\eta =\frac{\left(\Delta H_{M/caps}+\Delta H_{F/caps}\right)\times \Delta H_{M/PCM}}{\left(\Delta H_{M/PCM}+\Delta H_{F/PCM}\right)\times \Delta H_{M/caps}}\times 100\%,$$

The *η* values for the prepared microcapsules were in the 98–99% range. This implies an absence of confinement effects on the phase transitions of encapsulated *n*-Octadecane that is PCM within the capsules is involved in storage and release of thermal energy. This can be considered as a benefit of microencapsulated PCMs as it was reported previously that confinement restrictions may affect the phase transitions in nanoencapsulated PCMs and reduce their thermal storage capability [46,47,51]. However, electron miscopy revealed there was enough unoccupied volume within the prepared PU capsules, apparently, due to toluene evaporation. Thus, *n*-Octadecane had no restrictions to expand when heated and transformed to liquid phase.

**Figure 8:**
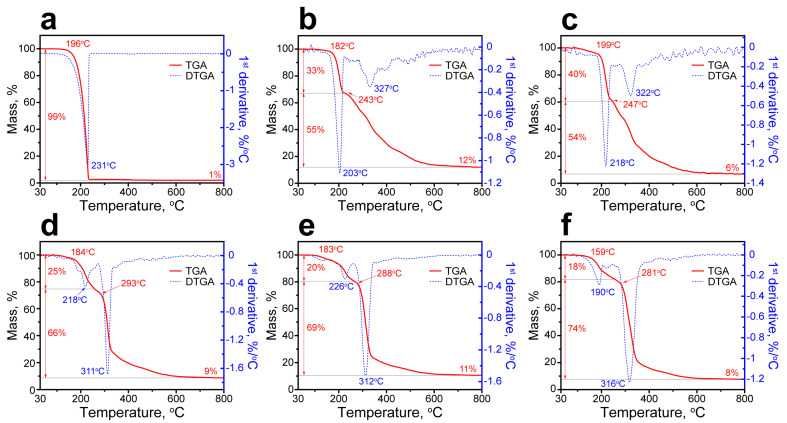
Thermal decomposition patterns of bare *n*-Octadecane (**a**), PU-1 (**b**), PU-2 (**c**), PU-3 (**d**), PU-4 (**e**), and PU-5 (**f**) capsules measured with TGA and DTGA.

Figure 8 shows the thermal decomposition patterns of the PU capsules and initial *n*-Octadecane acquired by TGA and DTGA measurements. The decomposition pattern of *n*-Octadecane had one stage with the onset temperature *T_on_* of 196 °C. The maximum rate of decomposition (MRDT) was at 231 °C, while the complete degradation was reached at 235 °C and attended with the total mass loss of 99% (Figure 8a). In the decomposition patterns of the PU capsules, two stages can be distinguished associated with the initial decomposition of the hard segments of polyurethane shell followed by the release and decomposition of the encapsulated paraffin along with the decomposition of the residual soft shell segments [52]. According to DSC, PU-1 and PU-2 capsules demonstrated a relatively low actual *n*-Octadecane content. Therefore, their decomposition patterns were mostly defined by the decomposition of the PU shell with the most prominent weight loss at the first stage (Figure 8b,c). PU-1 capsules started to decompose at 182 °C with the MRDT of 203 °C and the mass loss at this stage of 33%. PU-2 capsules started to decompose at 199 °C with the MRDT of 218 °C and the mass loss at the first stage of 40%. This temperature range corresponds to the rupture of urethane linkages in hard PU domains, which are less stable comparing to soft ones and start to decompose at lower temperature owing to their smaller molecular weight and association per the intramolecular hydrogen bonds [52]. The further decomposition of PU-1 and PU-2 capsules was attended with a gradual weight loss due to the degradation of the residual PEG polymeric chains and encapsulated *n*-Octadecane. The DTGA curves of PU-1 and PU-2 capsules had only minor peaks at 327 and 322 °C, indicating the simultaneous decomposition of the encapsulated paraffin and PU shell. At this stage, the mass loss of PU-1 and PU-2 capsules was 55% and 54% with a mass residue at 800 °C of 12 and 6%, respectively.

In contrast, the capsules with higher actual content of *n*-Octadecane and thinner shell demonstrated the most prominent weight loss related to the decomposition of the encapsulated paraffin at the second stage of degradation (Figure 8d–f). PU-3 capsules started to decompose at 184 °C with MRDT at the first stage of 218 °C and the mass loss of 25%. At the second stage, the decomposition started at 293 °C with MRDT of 311 °C and the mass loss of 66% (the residual mass at 800 °C was 9%). PU-4 capsules started to decompose at 183 °C with MRDT at the first stage of 226 °C and the weight loss of 20%. At the second stage, the decomposition started at 288 °C with MRDT of 312 °C and the mass loss of 69% (the residual mass at 800 °C was 11%). Finally, PU-5 capsules started to decompose at 159 °C with MRDT at the first stage of 190 °C and the mass loss of 18%. At the second stage, the decomposition started at 281 °C with MRDT of 316 °C and the mass loss of 74% (the residual mass at 800 °C was 8%). Unlike the PU-1 and PU-2 capsules, the DTGA curves of PU-3, PU-4, and PU-5 capsules had major peaks at the second stage of decomposition. This is in good agreement with the prominent growth of the actual *n*-Octadecane content in these capsules determined from DSC data. Although the PU capsules demonstrated a minor reduction in thermal stability compared to the bare paraffin, they still remained stable up to 180 °C, which much exceeds their expected working range as defined by the phase transition points.

Finally, the effect of capsule shell structure and *n*-Octadecane feeding during the capsule preparation was evaluated by incubation of the PU capsules in cyclohexane. The leakage rate was figured out with respect to the mass loss (*LR_m_*) as:

(6) $$LR_{m}=\frac{m_{i}-m}{m_{i}}\times 100\%,$$

where *m_i_* is the initial mass of the capsules, and m is the mass of the capsules after incubation. Additionally, the leakage rate was calculated regarding the actual content of *n*-Octadecane derived from DSC measurements as follows:

(7) $$LR_{E}=\frac{E_{a0}-E_{a}}{E_{a0}}\times 100\%,$$

where *E_a_*_0_ is the actual *n*-Octadecane content prior to the incubation and *E_a_* is the actual *n*-Octadecane content prior the incubation. The results of the leakage rate evaluation are summarized in Table 2.

According to Equations (6) and (7), the leakage rate of *n*-Octadecane gradually increased along with its actual content and reduction in capsule shell thickness. This confirms that the low feedings of *n*-Octadecane resulted in less PAPI segregation from the oil phase and, therefore, more isocyanate was involved in interfacial polymerization. Eventually, this led to a robust shell structure capable of preventing the paraffin extraction to continuous phase. On the other hand, the increase in *n*-Octadecane feeding led to thinner capsule shells as more PAPI was displaced from the oil phase at emulsion preparation and, thus, was not involved in interfacial polymerization. The calculated *LR_m_* and *LR_E_* values are in good agreement, although slightly different. This can be due to the DSC analyses being performed for a relatively small amount of the sample while, the mass measurements were carried out for the samples in whole.

### 3.3. Evaluation of Latent Heat Storage Properties in a Paint Composition

Summarizing the results of the previous parts of the work, the PU-3 capsules demonstrated the best combination of structural properties and latent heat capacity. Therefore, these capsules were chosen to be tested as a thermoregulating additive to the paint. To do this, the washed PU-3 capsules were dispersed in aqueous medium to obtain a concentrated capsule suspension, which was further added to the acrylic paint purchased from the local hardware shop. The supplier recommends diluting the initial paint with 5 wt% of water prior to use. However, we were required to exceed the recommended dilution to obtain a better capsule dispersion. The paint prepared in this way preserved good brushing quality and was brushed onto steel plates to determine the effect on their cooling rate. The thickness of the brushed layer was about 530 ± 50 nm. Additionally, free-standing layers of dried paint with PU-3 capsules were prepared to study the capsule distribution and integrity.

**Figure 9:**
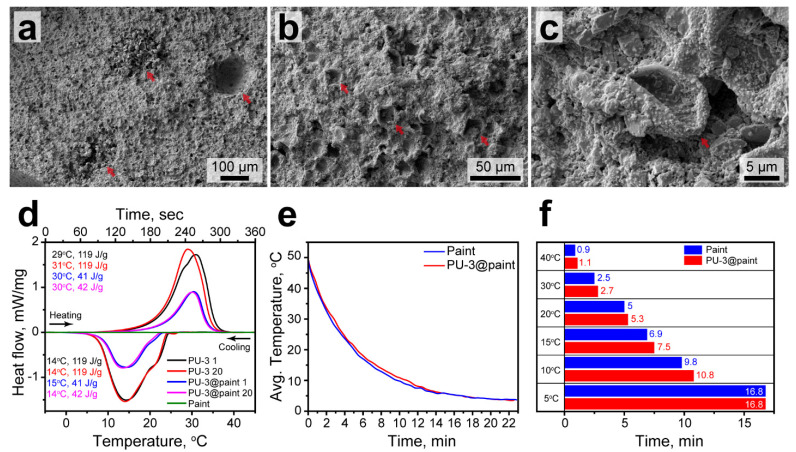
(**a**–**c**) The SEM images of the paint with addition of PU-3 capsules at various magnifications. The red arrows in (**a**) show the cavity in paint structure and capsule agglomerates. The red arrows in (**b**,**c**) show the PU-3 capsules in the paint structure; (**d**) heating and cooling curves of PU-3 capsules, the paint with PU-3 capsules, and initial paint. Indexes in the legend indicate a number of heating/cooling cycle; (**e**) cooling curves of steel plates brushed with the initial paint and the paint containing PU-3 capsules; (**f**) comparison of the cooling times of steel plates brushed with the initial paint and the paint containing PU-3 capsules at various temperatures.

Figure 9a–c show the SEM images of the paint layer with included PU-3 capsules. The capsule suspension was mixed well with the paint. The capsules were uniformly distributed within the pain layer, yet some capsule aggregates can be seen on low-magnification images (Figure 9a). This might be associated with the formation of air spaces in the paint layer due to manual blending. The cavities were subsequently filled with the capsules. The capsules can be clearly recognized within the paint by their distinctive curved morphology (Figure 9b). Taking a closer look, the capsules preserved their integrity after the paint solidification with no evidence of the broken shells (Figure 9c).

The latent heat capacity of the paint containing PU-3 capsules was evaluated with DSC and compared to this of the PU-3 capsules and the initial paint (Figure 9d). The heating and cooling curves of the paint with PU-3 capsules demonstrated the endothermic and exothermic peaks with the same melting and crystallization points as for PU-3 capsules (*T_M_* = 30 °C, *T_C_* = 14 °C). This means the inclusion of the capsules into the paint structure did not affect the phase transitions of the encapsulated *n*-Octadecane. Predictably, the latent heat capacity of the paint with addition of PU-3 capsules reduced toward the initial PU-3 capsules proportional to their content. The Δ*H_M_* and Δ*H_C_* values were 41 and 42 J/g. This gives the actual capsule content of 35% towards the PU-3 capsules and actual PCM content of 21% toward the bare *n*-Octadecane. This is consistent with some recent results on thermoregulating coatings with the addition of encapsulated PCMs. For instance, Qin et al. reported on the composite coating prepared by addition of SiO_2_ capsules with encapsulated *n*-Octadecane to thermoplastic acrylic resin [53]. In this coating, they reached the latent heat capacity of 71.35 J/g corresponding to 35% of the actual paraffin content. The bare capsules and capsules in the coating demonstrated the same melting and crystallization points. Naikwadi et al. reported on the preparation of polymeric capsules with encapsulated *n*-Docosane, which were introduced into acrylic-based paint [54]. The paint showed latent heat capacity of 35 J/g and 42 J/g after the addition of 50 wt% and 60 wt% of the capsules, respectively. In this study, the capsules embedded in paint also demonstrate the same phase transition points as the bare capsules. It should be noted that the laten heat capacity of the paint reported in our study can be further improved by employing PU-4 or PU-5 capsules with higher actual content of PCM.

The cyclic DSC measurements revealed good durability in latent heat capacity and thermal properties of the paint with PU-3 capsules. In 20 melting and crystallization cycles, the position and shape of endothermic and exothermic peaks did not demonstrate any prominent changes. The same is held for PU-3 capsules as well, however the endothermic peak on the melting curve shifted after the first melting/crystallization cycle. This can be explained by the change in the *n*-Octadecane conformation within the capsules. Additionally, Figure 9d demonstrates the heating and cooling curves of the initial paint without the addition of PU-3 capsules. There were no peaks on the curves, which means the paint did not have a latent heat capacity itself in this temperature range.

Finally, the effect of PU-3 capsules on the cooling rate of paint brushed on steel plates was evaluated with infrared thermography. The averaged cooling curves of steel plate brushed with initial paint and paint with addition of PU-3 capsules measured in the temperature range from 50 to 5 °C are shown in Figure 9e. The cooling curves are mostly defined by the cooling behaviour of steel substrates. However, the plate brushed with the pain containing PU-3 capsules cooled slightly slower. This was the most prominent in the 20–10 °C range, corresponding to the exothermic peak of the paint with PU-3 capsules according to DSC. The comparison of cooling times shows that the addition of PU-3 capsules to paint slowed down the cooling of the painted surface to 15 °C by 0.6 min and to 10 °C by 1 min.

These preliminary studies confirmed that the paraffin-loaded PU capsules have a potential to be employed as an additive to the commonly used paints to improve their latent heat storage properties and thermoregulating performance. The extended cooling time implies the paint is capable to accumulate the excess of external thermal energy with it, following prolonged release. Thus, the maintenance of the comfortable indoor temperature potentially will demand less fuel consumption for heating. The heat can be accumulated whether from central heating or from abundant renewable like the sunlight in the daytime. In this study, we have employed *n*-Octadecane as a model paraffin PCM. Further studies may be devoted to the adaptation of the capsule working temperatures to the human comfort range. The adjusting of core-to-monomer ratio and PCM-to-solvent ratio to increase the latent heat capacity and optimize the PAPI consumption are also of particular interest.

## 4. Conclusions

In this work, we have demonstrated the preparation of shape-stable polyurethane capsules loaded with *n*-Octadecane via polymerization of commercially available polymethylene polyphenylene isocyanate (PAPI) and PEG 1000 at the oil/water interface of PVA-stabilized emulsion. The successful shell polymerization and encapsulation of *n*-Octadecane were proved with FTIR spectroscopy and confocal microscopy. Electron microscopy revealed the formation of micron-sized (9.4–16.7 µm) quasi-spherical capsules with a smooth outer surface and curved inward morphology. Additionally, SEM established that the thickness of the resulted PU shell reduced from 460 to 220 nm and the feeding of *n*-Octadecane to the emulsion oil phase increased from 1.6 to 8 g at the same quantity of solvent. This can be related to the reduction in PAPI solubility, leading to its segregation from the oil phase. Nevertheless, TEM confirmed the paraffin is reliably encapsulated by PU shell and free to undergo the phase transition in the capsule interior.

The prepared PU capsules had tuneable latent heat capacity (51–133 J/g) defined by *n*-Octadecane feeding in the oil phase. Furthermore, the latent heat capacity and phase transition points remained stable at least for 20 melting/crystallization cycles, as exampled by PU-3 capsules. According to DSC, the actual *n*-Octadecane content was found to increase gradually along with the mass fraction of paraffin in the oil phase until it reached 6.4 g. The further increase in the *n*-Octadecane feeding to 8 g did not result in the significant improvement in the latent heat capacity. The TGA analysis revealed the capsules with various actual *n*-Octadecane content demonstrated different thermal decomposition patterns. In capsules with relatively low actual *n*-Octadecane content (PU-1 and PU-2), the most prominent impact was found due to decomposition of the PU shell. On the other hand, in capsules with higher actual *n*-Octadecane content (PU-3, PU-4, and PU-5), the most prominent mass loss was due to the decomposition of encapsulated paraffin. This is in good agreement with DSC data and PU shell thickness obtained from SEM images. Importantly, the capsules remained stable in the working temperature range of *n*-Octadecane.

The prepared PU capsules were feasible as thermoregulating additives to commercially available acrylic paints, as exampled by PU-3 capsules. The preliminary experiments demonstrated that the PU capsule content may reach up to 35 wt% in the composition. Overall, the capsules were well dispersed, although some agglomerates were found in SEM images. The reason for agglomeration is likely to be due to the manual mixing of a small paint volume. The addition of the PU-3 capsules improved the latent heat capacity of the paint to 41 J/g in the temperature range from 20 to 10 °C. This corresponded to the actual content of 21% toward the initial *n*-Octadecane. The latent heat capacity of the paint with PU-3 capsules remained stable within at least 20 melting/crystallization cycles. Finally, the addition of PU-3 capsules to the paint was found to slow down the cooling rate of the brushed steel plate in comparison to the initial paint without the capsules.

## Figures and Tables

**Table 1:** The compositions used for preparation *n*-Octadecane-loaded PU capsules.

Sample	*n*-Octadecane, g	Toluene, g	Monomers (PAPI + PEG), g	Core-to-Monomer Ratio (wt.)	*n*-Octadecane-to-Toluene Ratio (vol.)
PU-1	1.6	15.6	6.4	2.69	0.07
PU-2	3.2	15.6	6.4	2.94	0.14
PU-3	4.8	15.6	6.4	3.19	0.21
PU-4	6.4	15.6	6.4	3.44	0.28
PU-5	8	15.6	6.4	3.69	0.35

**Table 2:** The leakage rate of *n*-Octadecane from the PU capsules after incubation for 24 h in cyclohexane.

Sample	*m_i_*, mg	*m*, mg	*LR_m_*, %	*E_a_*_0_, %	*E_a_*, %	*LR_E_*, %
PU-1	101.0	97.6	3.4	26.6	26.3	1.1
PU-2	100.2	94.1	6.1	34.4	32.6	5.3
PU-3	100.6	91.3	9.2	60.5	53.9	10.9
PU-4	100.0	81.4	18.6	66.6	56.9	14.5
PU-5	100.8	63.7	36.8	67.2	44.3	34.1

## Data Availability

The data that support the findings of this study are available from the corresponding author upon reasonable request.

## Supplementary Materials

The following supporting information can be downloaded at: https://www.mdpi.com/article/10.3390/ma16196460/s1, Video S1: Melting of encapsulated *n*-Octadecane within the PU-2 capsule interior.
